# Advances and applications in medical coating materials: a comprehensive review

**DOI:** 10.3389/fchem.2026.1760661

**Published:** 2026-02-20

**Authors:** Xinmeng Yang, Xiaoling Yu, Shuiwei Qiu

**Affiliations:** 1 Heart Center, Women and Children’s Hospital, Qingdao University, Qingdao, Shandong, China; 2 Department of Infectious Diseases, Women and Children’s Hospital, Qingdao University, Qingdao, Shandong, China; 3 Department of Cardiothoracic Surgery, The Quzhou Affiliated Hospital of Wenzhou Medical University, Quzhou People’s Hospital, Quzhou, Zhejiang, China; 4 School of Medicine, Shaoxing University, Shaoxing, Zhejiang, China

**Keywords:** bioactivity, biocompatibility, medical-coating, surface modification, synergistic modification

## Abstract

This review synthesizes the current state of knowledge on medical-coating materials. Fundamental theories are examined with respect to material taxonomy, physicochemical properties, biocompatibility profiles, and antimicrobial mechanisms. Clinical applications are reviewed for implants, wound dressings, catheters, and endovascular stents. Technological advances are highlighted in nanotechnology, surface-modification strategies, and the development of stimulus-responsive “smart” coatings. The analysis traces the field’s evolution, noting its growing market and clinical adoption, while identifying persistent challenges in long-term biosafety and translation. Future directions are projected toward synergistic modification for multifunctionality, patient-specific designs, and next-generation intelligent systems. By integrating contemporary research, this review aims to inform and guide the future development and clinical application of advanced coating technologies.

## 1 Introduction

Biomedical implants often come into direct contact with human tissues or blood after implantation, posing potential safety risks such as thrombosis, infection, rejection reactions, and inflammation (Wang et al., 2021). Applying medical coating materials for surface modification of these devices is an important strategy to overcome the limitations of the base materials. Medical coating materials are thin layers applied to the surfaces of medical devices or implants to regulate their interactions with biological tissues. The application of coating can not only enhance the physicochemical properties of materials and confer excellent biocompatibility, but also improve antibacterial performance, achieve local controlled drug delivery, and reduce adverse tissue reactions, among other functions (Rajesh et al., 2024; Wang et al., 2022; Yao et al., 2022). Currently, crucial research problems in this field include regulating the structure and composition on the surface of biomedical materials, constructing functional surfaces, and achieving low complication of diseases and higher biocompatibility. In recent years, with the rapid development of materials science, emerging concepts such as nanotechnology and smart responsiveness have gradually entered the stage of technological application. The field of medical coatings is rapidly advancing beyond traditional materials science into the realm of intelligent systems. However, many reviews remain siloed in disciplinary perspectives. In this work, we trace the trajectory from bio-inert to bioactive, and now to bio-responsive interfaces, by synthesizing insights from nanotechnology, polymer chemistry, and cell biology, offering a strategy to design the intelligent coating.

**FIGURE 1:**
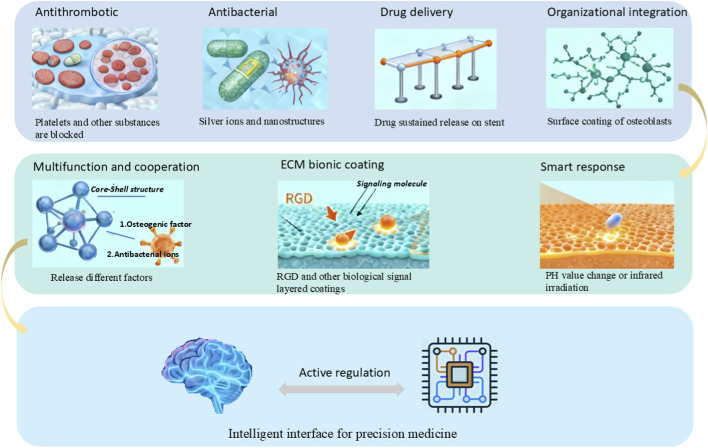
Schematic of the development and progress of medical coatings.

This narrative review aims to synthesize and critically discuss the latest research progress and clinical applications. By integrating findings from key studies, it provides a comprehensive overview and identifies emerging trends. It begins by classifying coating materials and detailing their fundamental characteristics. Next, it examines key functional categories—such as antithrombogenic, antimicrobial, drug-delivering, and tissue-integrating coatings—along with contemporary advances. The discussion then extends to emerging trends, including smart responsive systems, multifunctional designs, and biomimetic strategies. Finally, the review assesses current challenges and provides a forward-looking perspective on the future development of medical coatings (Figure 1).

## 2 Classification and characteristics of medical coating materials

### 2.1 Inorganic bio-coating

To overcome the limitations of traditional base materials, inorganic bio-coating have become an important direction for surface modification of medical materials due to their unique bioactivity and tunable functionality. Commonly used inorganic coating materials mainly include inorganic non-metallic materials and metallic materials. Inorganic non-metallic materials include silicates, ceramics, glass, etc., while new inorganic materials include silica aerogels, carbon nanotubes, etc. Taking orthopedic implant coatings as an example, the systematic review of various materials found that hydroxyapatite (HA) coating is still considered one of the most biocompatible coatings (Yao et al., 2025). HA is widely present in bone tissue and teeth of organisms, with the main component of Ca_10_(PO_4_)_6_(OH)_2_, possessing excellent biocompatibility and osteoinductivity (Sri and Usha, 2025). Depositing the HA coating on metal alloys can enable effective osseointegration between the implant and surrounding tissues. Metallic materials are commonly used inorganic materials in the field of material modification, with inert metals having excellent biocompatibility. Some researchers prepared a zirconium (Zr) coating on the surface of Ti-6Al-4V alloy using radio frequency magnetron sputtering and found that the surface energy of the Zr-coated group was significantly higher than that of the Ti-6Al-4V group (P < 0.05). Furthermore, in terms of cell proliferation, alkaline phosphatase (ALP) activity, and the expression of bone sialoprotein (BSP) and osteocalcin (OCN) genes, the Zr-coated group performed better, which indicate that the Zr coating can enhance early osteoblast response and is more suitable for orthopedic and dental implants (Lee et al., 2014). Magnesium alloys and iron alloys are widely used in chemistry, microelectronics, aerospace, biomedicine, and other fields due to their high specific strength, good electromagnetic shielding, strong biocompatibility, excellent degradability, and antibacterial properties. However, due to the chemically active nature of these metals, they easily form oxides or hydroxides upon contact with tissues, which severely affects their service life in complex environments. To enhance the corrosion resistance of alloys in complex environments such as the human body, in addition to the aforementioned surface modification of metallic materials, novel inorganic materials represent a promising direction for implant coatings. Zhang et al. used calcium silicate and hydroxyapatite as coating materials for bone repair scaffolds. Their mechanical performance studies indicated that the coating material could effectively fill the pores of the porous scaffold, with increasing the average compressive strength of the scaffold by 55%. The higher the calcium silicate content in the coating, the faster the degradation rate, that achieved controllability of the repair scaffold’s degradation rate. Cell experiments demonstrated that scaffolds coated with calcium silicate-rich coatings exhibited better cytocompatibility, facilitating cell proliferation and differentiation (Zhang et al., 2021). The mechanism of inorganic molecular coatings modifying base materials primarily revolves around overcoming the chemical inertness and hydrophobicity of the base material surface by firmly attaching target inorganic molecules to the surface, thereby endowing it with new surface functions: bioactivity, antibacterial properties, conductivity, wear resistance, hydrophilicity, etc. Specifically, inorganic molecular coatings function through one or more of the following pathways: first, by releasing ions to regulate cell metabolism and promote tissue differentiation (Abualia et al., 2024); second, by constructing rough or porous structures to increase the specific surface area and provide cell anchoring sites (Zhao M. Y. et al., 2025); third, by improving surface hydrophilicity to promote cell adhesion, proliferation, and differentiation (Liu et al., 2024). According to various application needs, medical coating materials are diverse, exhibiting different characteristics. HA and its derivatives, due to their identical composition with the primary inorganic components of bone matrix, directly promote new bone deposition through osteoconduction (Xu et al., 2025). Sol-gel materials like silica and titania can enhance antibacterial properties and achieve good biocompatibility through ion release (Barrino, 2024).

### 2.2 Polymer coatings

Synthetic polymer materials have become an indispensable core material in the field of medical coatings due to their wide tunability, excellent processing performance, and diverse biological functions. Synthetic polymers can significantly improve the interaction between medical devices and the host tissue environment, endowing them with functions such as anticoagulation, antibacterial properties, promotion of cell adhesion, and drug delivery. There are various types of synthetic polymer materials, among which non degradable materials mainly provide long-lasting surface functionality for medical materials. For example, polyurethane, with excellent toughness and blood compatibility, is widely used in artificial blood vessels and other substitute materials; polytetrafluoroethylene (PTFE), with very low friction coefficients and anti-cell adhesion capabilities, are often coated on stents and drainage catheters; polyethylene glycol (PEG) and its derivatives can effectively resist non-specific protein adsorption and bacterial or cell adhesion through their unique high hydrophilicity and “spatial repulsion”, and are commonly used to construct antibacterial coatings. Degradable polymer materials can hydrolyze into small molecules in the body after performing specific functions, which are then excreted through body metabolism, they were mainly used for temporary barriers and controlled drug release systems. For instance, polylactic acid (PLA), polyglycolic acid (PGA), etc., are currently the most widely used bio-coating materials, with adjustable degradation rates, widely used on drug-eluting stent surfaces; while polycaprolactone (PCL) is often used in long-term drug delivery systems with a longer degradation time and good drug permeability; the surface erosion characteristics of poly (lactic-co-glycolic acid) (PLGA) allow it to achieve near-zero-order drug release, primarily used for local chemotherapy.

On the other hand, natural biopolymer coatings used to modify medical materials, such as collagen (Col), gelatin (Gel), chitosan (CS), sodium alginate (SA), and fibrin, are used to improve cell adhesion, proliferation, and differentiation by regulating chemical groups and surface charge on the material surface. Studies have shown that immobilizing bioactive molecules on the material surface via polymer chains can effectively promote the adsorption of specific proteins while inhibiting non-specific protein adsorption (Chen, 2017). Gelatin is a partial hydrolysate of collagen and is one of the main components of the extracellular matrix. Processed gelatin has further reduced immunogenicity. Gelatin retains the Arg-Gly-Asp (RGD) sequence in collagen protein, which is a recognition site for integrin proteins and can directly mediate cell adhesion, a key advantage that many synthetic materials lack. Furthermore, active groups such as amino and carboxyl groups on natural molecules like gelatin can conveniently bind with cross-linkers (like glutaraldehyde, genipin) or other functional molecules (like growth factors, drugs) to regulate their mechanical properties, degradation rate, and biological functions. Farnaghi et al. (2025) coated a gelatin layer on the surface of a polycaprolactone/hydroxyapatite nanocomposite and cross-linked it with EDC/NHS to improve the coating strength of the scaffold. The mechanical properties of the scaffold were significantly enhanced, proposing improved biodegradability and bioactivity. Furthermore, the gelatin-coated scaffold showed improved cell viability and adhesion, with the expression of OPN and ALP genes more than doubled, indicating improved biological characteristics. Bioactive glass is a standard biomaterial used for hard tissue regeneration in orthopedics and dentistry. Due to its significant characteristic of releasing calcium ions, bioactive glass has good osteoinductivity. However, this release is not easily controlled and may often be excessive, especially during the initial interaction stage between the biomaterial and surrounding tissues. In one study, researchers tested chitosan biopolymer coatings of different thicknesses to regulate the calcium ion release rate from bioactive glass nanoparticles, and achieve good results. As the chitosan concentration in the coating solution increased, the calcium released from the chitosan-coated particles gradually decreased (Uskokovic et al., 2024). Natural biopolymer coatings like chitosan, possessing good compatibility, can carry antibacterial agents (e.g., silver nanoparticles) (Huang et al., 2023), growth factors (e.g., Epidermal Growth Factor, EGF), or antioxidants (Gou et al., 2024), achieving multifunctionalization such as antibacterial, anti-inflammatory, and pro-healing effects.

## 3 Functional classification and research progress of medical coatings

### 3.1 Antithrombogenic and hemocompatible coatings

Preventing thrombosis and possessing good hemocompatibility are fundamental requirements for cardiovascular devices (stents, catheters, valves, etc.). Currently, heparin-based coatings for thrombosis prevention, whether deployed via covalent immobilization or layer-by-layer self-assembly techniques, remain the critical clinical method. The research and development of heparin anticoagulant coating technology began in the 1970s and 1980s, and heparin coatings gradually entered commercial application in the 1990s. Now, various heparin coatings such as Trillium biosurface, Bioline coating, and Corline heparin surface (CHS) are used clinically (Yan and Yan, 2023). The Carmeda bioactive surface (CBAS heparin surface), developed by the Swedish company Carmeda AB, is one of the most widely used heparin coating technologies in the world with the most approved products. The CBAS coating has a unique “end-point attachment” (EPA) method, which uses terminal groups as active sites to covalently fix the long heparin molecular chain onto the material surface. Gore et al. (2014) in 2014 verified the advantages of the “end-point attachment” method over other fixation methods; EPA allows heparin to retain its activity better, resulting in superior anticoagulant effects. Extensive clinical trial data have validated the safety and efficacy of heparin anticoagulation technology, but some shortcomings remain, such as relatively low heparin activity and single functionality. Medical devices like artificial hearts, artificial valves, and artificial blood vessels that are in long-term contact with blood require additional functional needs besides anticoagulation, such as antibacterial properties and anti-inflammatory responses (Huang et al., 2024; Wang et al., 2025). Current heparin coating performance cannot fully meet these requirements. Additionally, some reports indicate that heparin coatings can also have certain side effects, such as heparin-induced thrombocytopenia (HIT) (THAKUR et al., 2009).

**FIGURE 2:**
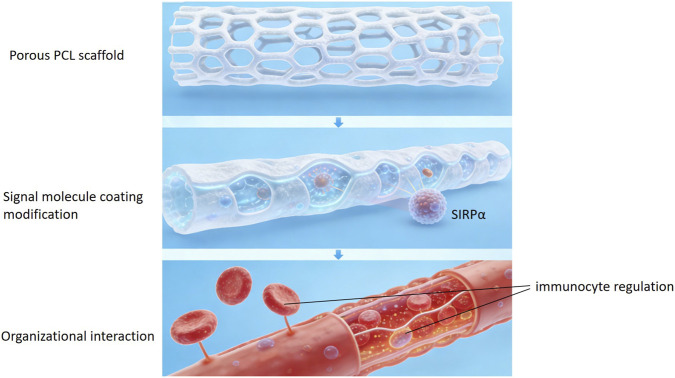
CD47-SIRP α surface modified porous PCL scaffold.

The human cell membrane is composed of a phospholipid bilayer, with its hydrophilic “head” groups facing the aqueous environment on both sides and the hydrophobic “tail” groups facing the interior of the membrane. On the outer surface of the cell membrane, phosphatidylcholine is one of the most abundant phospholipid molecules. Using phospholipid polymers via a biomimetic approach can effectively reduce protein adsorption and platelet adhesion. Poly (2-methacryloyloxyethyl phosphorylcholine), abbreviated as PMPC, has side chains containing phosphorylcholine groups identical to those in natural phosphatidylcholine. When PMPC is coated or grafted onto a material surface, the phosphorylcholine groups can bind a large number of water molecules through hydrogen bonding, forming a highly ordered, stable “hydration layer,” creating a physical steric hindrance and energy barrier, thereby inhibiting protein adsorption, preventing cell adhesion, and producing an antithrombotic effect. Wang et al. (2023) designed and synthesized a multifunctional coating material using lysozyme (Lyso) and PMPC. By reducing the disulfide bonds in Lyso-PMPC, a new nanofilm (PTL-PMPC) was obtained. The new film exhibited excellent anti-fouling performance against cells, bacteria, fungi, proteins, biological fluids, phosphates, polysaccharides, esters, and carbohydrates. Furthermore, this coating showed good hemocompatibility and low cytotoxicity. In the development of blood-contact medical devices, careful design of the material surface is crucial. These materials not only need to possess excellent mechanical properties and stability but also should have outstanding hemocompatibility to resist thrombosis and inflammatory responses. Porous polycaprolactone (PCL) scaffolds prepared by fused deposition modeling reveal application potential in fields such as vascular grafts due to their controllable three-dimensional structure and adjustable mechanical properties. However, PCL material itself is hydrophobic, and when its surface contacts blood, it non-specifically adsorbs a large amount of plasma protein, subsequently activating platelets and the coagulation cascade, leading to thrombosis and implantation failure. In order to overcome this bottleneck, functionalizing the material surface utilizing the CD47-signal regulatory protein α (SIRPα) signal transduction pathway has become a cutting-edge bioengineering strategy (Tengood et al., 2016). By immobilizing CD47 or its mimetic peptides on the material surface, abnormal interactions between the material and immune cells in the blood can be actively regulated. This biomimetic modification can effectively inhibit platelet activation and aggregation, while reducing the inflammatory response caused by monocytes/macrophages, thereby significantly improving the blood compatibility of the material and providing a new direction for the development of a new generation of antithrombotic implantable devices (Figure 2).

### 3.2 Antibacterial and anti-infective coatings

According to statistics, the incidence of hospital-acquired infections caused by bacterial colonization on medical material surfaces is about 4%–10%. The core of medical device-related infections is the formation of bacterial biofilms, which occurs when bacteria undergo irreversible adhesion to the device surface. Therefore, inhibiting bacterial colonization and biofilm formation on medical device surfaces is a key problem that urgently needs solving. The design concept of modern antibacterial and anti-infective coatings has shifted from “simple sterilization” to “multi-strategy synergy”. Firstly, silver (Ag) remains one of the most widely used antibacterial agents in coating materials. Releasing silver ions (Ag^+^) through the device coating destroys bacterial cell membranes, interferes with enzyme systems, and damages DNA to achieve antibacterial effects. Nano-silver ions can significantly increase the specific surface area and antibacterial efficiency and can be loaded onto matrices like gelatin and chitosan to achieve sustained release (Alven and Aderibigbe, 2024; Halder et al., 2011; Li et al., 2025). In the field of antibacterial fabric coatings, poly (allylamine hydrochloride)-ZnO coating can be applied to cotton, nylon, and polyester fabrics. Relevant research results (Mlinaric et al., 2025) reported PAH/ZnO coatings containing rod-shaped ZnO nanoparticles on cotton fabrics reduced the cell viability of *staphylococcus aureus* by more than 99%, demonstrating good potential for wound dressing applications. In the field of antibacterial research achieved through physical and energy barriers, hydrophilic/hydrogel coatings, such as polyethylene glycol (PEG), zwitterionic polymers (e.g., poly (sulfobetaine)), etc., form a “hydration layer” on the substrate material surface through strong hydration, making it difficult for bacteria to colonize and adhere. In a recent study by Ande et al., the effect of different PEG concentrations (0.1 g, 0.3 g, and 0.5 g) combined with ZnO on antibacterial efficacy was systematically studied. Samples prepared with 0.3 g PEG showed higher ROS generation, indicating higher antibacterial activity. The experimental material exhibited broad antibacterial effects against *Staphylococcus*, *Bacillus*, *Klebsiella*, and *E. coli* (Ande and Reddy, 2025). Furthermore, superhydrophobic coatings can achieve antibacterial and anti-infective effects by constructing micro-nano composite rough structures on the substrate surface, forming a high contact angle, making it difficult for droplets or bacteria and other contaminants to attach (Jiang et al., 2019).

### 3.3 Drug delivery coatings

**FIGURE 3:**
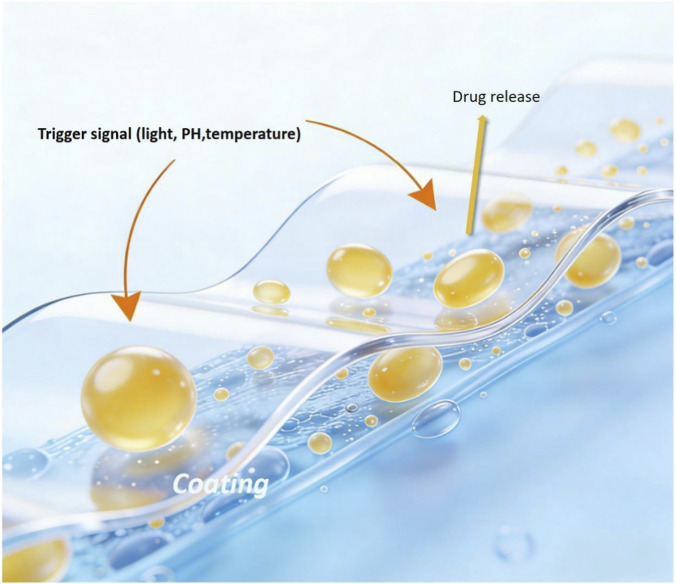
Drug release coating system.

Drug delivery coatings are one of the most successful and influential branches in the field of medical coatings (Figure 3). Their core value lies in achieving localized and controlled drug delivery, thereby solving the inherent drawbacks of traditional systemic administration. They increase local drug concentration, directly releasing high doses of drugs at the lesion site, ensuring efficacy; reduce systemic side effects, greatly decreasing the amount of drug in the circulatory system, avoiding damage to non-target organs. Drug delivery coatings can enable drugs to take effect continuously for weeks or even months through sustained-release technology, improving patient compliance. Such coatings are usually composed of carrier materials and active drugs, and their design key lies in precisely controlling the drug release kinetics to match the pathophysiological processes of specific diseases. Drug coatings are widely used in drug-eluting stents (DES). Drug-eluting stents effectively address the problem of local coronary restenosis by coating the metal stent surface with a polymer layer containing anti-proliferative drugs, locally inhibiting smooth muscle cell proliferation. Their development has progressed from first-generation sirolimus-eluting stents using permanent polymers to new-generation stents using biodegradable polymer matrices (like PLGA) and new anti-proliferative drugs (like zotarolimus, everolimus). Beyond cardiology, coatings for cancer implants releasing chemotherapeutic drugs (like doxorubicin) and coatings for tissue engineering scaffolds delivering growth factors (like BMP-2 for bone regeneration) are being actively researched.

Since the 1990s, the vigorous development of nanotechnology has brought new changes to drug delivery methods. Because nanoparticles have characteristics such as high specific surface area and strong adsorption capacity, the loading capacity of drug molecules in nanocarriers is greatly increased; meanwhile, due to the small particle size of nanodrug carriers (5–200 nm), they can enter capillaries, flow freely in the blood circulation system, enter cells, and then be absorbed by cells and tissues through endocytosis, improving drug utilization. Mesoporous silica nanomaterials have found many applications in nanodrug carriers due to their highly ordered, tunable pore channels, large pore volume, adjustable pore size, extremely high specific surface area, ease of functionalization, and good biocompatibility. Vallet-Regi et al. (2001) first used silica nanoparticles as a drug delivery system in 2001, investigating the effect of material pore size on the ibuprofen release rate using ibuprofen as a drug model. Lee’s team (Lee et al., 2024) overcame the traditional limitations of drug specificity by modifying mesoporous silicon materials with carboxymethyl cellulose (pSiNPs-CMC), etc. Wang N. et al. (2020) directly coated drug crystals with silica, significantly improving drug loading and expanding the application scenarios of silicon materials. More and more scholars are researching controlled release systems for drugs using nanoparticles like mesoporous silica, widely applied in numerous fields such as nanotechnology, materials science, and clinical medicine.

### 3.4 Tissue integration and regenerative coatings

**FIGURE 4:**
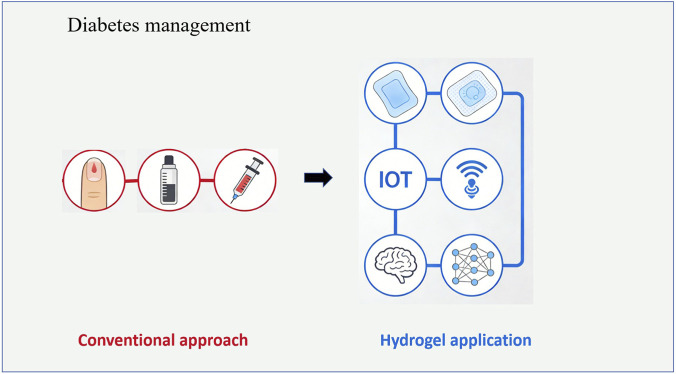
IoT combined with hydrogel technology to achieve a new management model for diabetes.

The application of coating materials in wound dressings aims to promote wound healing and prevent infection. Taking chlorhexidine hexametaphosphate (CHX-HMP) as an example, the Barbour team studied its antibacterial efficacy, toxicity, and effect on healing as a coating for wound care materials (Barbour et al., 2026). Experiments comparing CHX-HMP with chlorhexidine digluconate materials found that CHX-HMP coated materials exhibited antibacterial efficacy on days 1, 3, and 7. The toxicity to human placental cells was lower than that of commercial chlorhexidine dressings, and when used in Pluronic gel, they did not cause delayed healing and reduced the probability of wound colonization by *Enterococcus faecalis*. In tissue regeneration applications, biopolymer hydrogels are used as coating materials for wound care and diabetes management due to their properties like high water absorbency (Asl et al., 2024). In diabetes management (Figure 4), Non-invasive hydrogel technology offers the possibility to replace traditional methods. Combined with the Internet of Things (IoT) and machine learning systems, it holds promise for more effective diabetes diagnosis, management, and treatment (Gao and You, 2021). These coatings promote stable and functional connections between implants and host tissues.

Furthermore, in the field of tissue regeneration, besides chemical composition, surface physical structures, such as morphology and roughness, also have profound effects on cell behavior. Some scholars create micro/nano-scale topographies by manufacturing fiber-like, porous, or groove-like structures on the coating surface that mimic the natural ECM, which can guide directional cell migration and differentiation. In terms of improving biocompatibility, modifying the material surface to mimic the composition and structure of the extracellular matrix promotes cell-material interactions and reduces immune responses. For example, Chen et al. (2015) supported stem cell osteogenic differentiation by embedding a hyaluronic acid/β-tricalcium phosphate (HA/TCP) matrix into PCL scaffolds. On day 21, the DNA amount in the HA/TCP-coated scaffold was significantly higher. At the gene expression level, the HA/TCP coating significantly increased the expression of ALP and COL I on day 4. The HA/TCP coating significantly improved cell proliferation, osteogenic differentiation, and uniform distribution of the cell matrix. In a study by Kim et al. (2021), a 3D scaffold coated with exosome-coated silk fibroin (SF) successfully enhanced bone regeneration in rat calvarial defect areas by mimicking the matrix environment. The exosome-coated SF scaffold had beneficial effects on the growth and osteogenic differentiation of human bone marrow mesenchymal stem cells (hBMSCs). The aforementioned studies reveal that medical coatings can be biofunctionalized with extracellular matrix (ECM)-derived peptides (like RGD, YIGSR) or proteins to achieve specific cellular responses, promoting faster and stronger tissue integration.

### 3.5 Smart and responsive coatings

Smart responsive coatings aim to achieve more precise and efficient control. They can perceive specific physiological or pathological signals at the implantation site (endogenous stimuli, such as pH, enzymes, reactive oxygen species) or externally applied trigger signals (exogenous stimuli, such as light, magnetism, temperature), and use these to regulate their physicochemical properties, achieving on-demand drug release, dynamic switching of bioactivity, or real-time regulation of surface functions (Chen et al., 2024). Traditional medical coatings primarily provide passive biocompatibility or single drug sustained-release functions; their behavior is preset and unchangeable, unable to adapt to the complex, variable, and dynamic physiological and pathological environment in the body. This leads to issues such as premature drug release, insufficient dosage, or overdose (Zhao Y. et al., 2025). The microenvironment inside biofilms is usually weakly acidic. Coatings can be designed to degrade or undergo charge reversal under acidic conditions, releasing encapsulated antibiotics or nanoparticles. Magnetic nanoparticles (MNPs), due to their biocompatibility and superparamagnetism, are often used as drug carriers. Functionalizing MNPs with different surface coatings can achieve pH or temperature sensitive drug loading and targeted release, while external magnetic fields can guide them to the desired pathological area, reducing drug side effects on normal tissues (Aslam et al., 2022). When preparing nanocarriers for brain drug delivery, the physicochemical properties and surface modification of the nanocarriers are crucial for crossing the blood-brain barrier. Modifying the chemical structure of nanocarriers through surface coating strategies can regulate their interaction with brain endothelial cell membranes, such as attaching targeting ligands to achieve drug delivery to specific parts of the brain (Alotaibi et al., 2021). In terms of antibacterial materials, smart antibacterial coatings provide accessibility for effectively killing viruses and bacteria and reducing disease transmission (Dehghani et al., 2023). Currently, materials for smart antibacterial coatings mainly include copper, silver, titanium dioxide, silicon nitride, etc., among which titanium dioxide is one of the most widely used antibacterial substances. Titanium dioxide utilizes ultraviolet radiation to decompose organic matter in the air, producing oxides and free radicals, thereby achieving sterilization, deodorization, and air purification effects (Felice et al., 2017; Miao et al., 2021). In cutting-edge applications of smart coatings, for smart drug delivery systems in cancer treatment, thermo/pH-triggered hollow mesoporous carbon nanocarriers (PNIPAM@HMCs) were prepared, synthesized by a template method and coated with a pH/thermo dual-responsive poly (N-isopropylacrylamide) (PNIPAM) shell, enabling thermotherapy and drug release under near-infrared (NIR) radiation. NIR results showed a significant increase in ΔT for PNIPAM@HMCs, and a model based on small-angle X-ray scattering (SAXS) simulations verified the drug release kinetics, providing an effective strategy for cancer treatment (Lin et al., 2023).

Furthermore, the preparation of smart antibacterial coatings is also an important part of research and development. Currently commonly used coating preparation techniques include physical vapor deposition, chemical vapor deposition, sputtering technology, chemical solution methods, etc. Different preparation processes significantly impact the coating’s performance and antibacterial effect (Chen et al., 2024; Koosha and Hamedi, 2019). Physical vapor deposition technology, such as pulsed laser deposition used to prepare silver-doped hydroxyapatite coatings, can form nanocrystalline structures, significantly improving the sustained release kinetics of silver ions, thereby exhibiting long-term antibacterial activity superior to traditional plasma-sprayed coatings (Jelínek et al., 2010). The complementary chemical vapor deposition technology is adept at preparing ultra-thin, dense, and uniform films, for example, forming diamond-like carbon films on device surfaces, which themselves have excellent biocompatibility and anti-adhesion properties. If elements like silver or zinc are introduced during this process, a balance between excellent antibacterial performance and low cytotoxicity can be achieved (Zia et al., 2023). Magnetron sputtering technology has unique advantages in preparing multilayer nanostructures. By precisely controlling the thickness of each layer and element doping, composite coatings such as SiO_2_/Ag can be prepared. This coating can achieve efficient antibacterial effects with good cytocompatibility by precisely controlling the low-concentration release of Ag^+^, effectively resolving the contradiction between antibacterial properties and biosafety. In contrast, chemical solution methods (like sol-gel, layer-by-layer self-assembly) offer flexibility for molecular-level design under mild conditions. The quaternary ammonium salt-phosphate bifunctional coating developed using this method not only achieves high bonding strength on the titanium surface through covalent bonding but also efficiently destroys bacterial cell membranes through its positively charged quaternary ammonium groups, achieving “contact” sterilization and avoiding the drug resistance problems caused by antibiotic abuse (Ohta et al., 2008; Pan et al., 2022). In summary, selecting appropriate preparation techniques is key to realizing the specific antibacterial mechanisms and excellent comprehensive performance of coatings. From physical barriers and metal ion release to active contact sterilization via chemical solution methods, technological progress continuously promotes the development of antibacterial coatings towards high efficiency, safety, and multifunctionality.

## 4 Emerging trends in medical coating materials

With the deep integration of basic science and engineering technology, research on medical coatings is rapidly developing beyond the traditional single-function model towards integration, intelligence, and humanization.

### 4.1 Multifunctional and synergistic coatings

**FIGURE 5:**
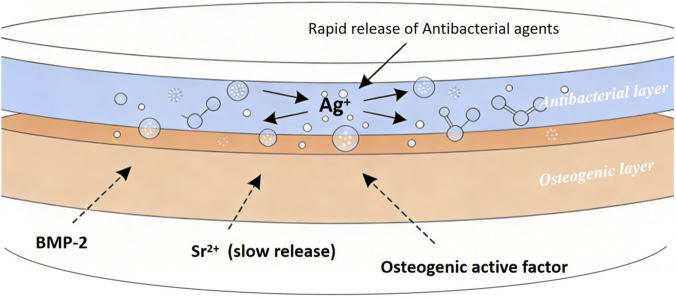
Antibacterial-osteogenic synergistic coating: multifunctions integrate into a single system.

Integrating multiple functions into a single coating system has become a mainstream strategy for coping with complex *in vivo* environments. The core of this trend lies in achieving synergy and complementarity between different functional modules through ingenious material design, rather than simple superposition. Traditional antibacterial coatings (e.g., releasing silver ions or antibiotics) may simultaneously kill pathogenic bacteria and produce toxicity to surrounding tissue cells (e.g., osteoblasts), inhibiting the osseointegration process. In contrast, antibacterial-osteogenic synergistic coatings are designed with bilayer or core-shell structures having different release kinetics (Figure 5). This allows the material surface layer to rapidly release antibacterial agents to prevent early infection; the inner layer slowly releases osteogenic bioactive factors (like BMP-2, Sr^2+^) to promote mid-to-long-term bone healing, reducing impact on autologous tissue (Pagani et al., 2025). Additionally, using certain ions (Zn^2+^) or molecules (antimicrobial peptides) that simultaneously possess mild antibacterial activity and the ability to stimulate osteogenic differentiation avoids functional conflict at the source, also achieving synergistic and complementary effects (Liu et al., 2018; Wang Z. T. et al., 2020).

### 4.2 Biomimetic and ECM-mimetic coatings

Currently, the development of coating materials has surpassed simple biomolecule immobilization. Cutting-edge research is committed to comprehensively simulating the complex characteristics of the natural extracellular matrix from the molecular to the microscopic scale, providing cells with an environment closest to the *in vivo* state. For example, Gu et al. (2023) used a biochemical signal biomimetic pathway, employing RGD-coated responsive 3D polymer extracellular matrix systems to enrich cancer stem cells, sustain stemness, and avoid enzymatic dissociation. Further development includes “multi-signal” interfaces containing various cell-specific ligands (e.g., YIGSR for endothelial cells, IKVAV for nerve cells) to precisely guide the adhesion and screening of different cells (Hosseinkhani et al., 2013; Kuzminska et al., 2021). Besides, using technologies like nanoimprinting and electrospinning, nanoscale grooves, pores, or fiber networks comparable in scale to natural ECM fibers can be manufactured on the coating surface. These topological structures can directly regulate cell adhesion, spreading, migration, and even stemness maintenance through the “contact guidance” effect. They can match the coating’s elastic modulus with the target tissue (e.g., soft brain tissue, hard bone tissue) by regulating cross-linking density or material selection (Wang et al., 2018). Cells can perceive substrate stiffness and differentiate accordingly; for example, mesenchymal stem cells are more likely to differentiate into osteoblasts on coatings simulating bone hardness.

### 4.3 Porous scaffold and hydrogel coatings

The application scope of coating technology has gradually expanded from planar implant surfaces to the interior of three-dimensional porous scaffolds or hydrogels, aiming to create a three-dimensional, bioactive support structure for tissue regeneration. Internal surface functionalization is achieved by methods such as physical adsorption, covalent grafting, or biomimetic mineralization, uniformly coating bioactive layers on the intricate internal pore walls of the scaffold. This allows cells growing deep into the scaffold to also receive signals promoting proliferation and differentiation. Hydrogels serve as injectable active coatings, encapsulating cells and growth factors in smart hydrogels and injecting them into the defect site (Faulk et al., 2014). This hydrogel itself is a three-dimensional, biodegradable temporary ECM that can dynamically regulate its physical properties and signal release in response to the environment (e.g., enzymes, pH), guiding orderly tissue regeneration.

## 5 Clinical translation and studies

In the cardiovascular field, long-term clinical studies of drug-eluting stents (DES) and heparin coatings have established their cornerstone position in reducing the risk of restenosis and thrombosis (Biran and Pond, 2017; Gogas et al., 2014). In the fields of orthopedics and dentistry, have been validated for decades for their bone integration efficacy, while antibacterial coatings loaded with have shown clear effects in reducing early infection rates in high-risk wound implants (Phogat and Awasthi, 2025). Emerging antibacterial osteogenic synergistic coatings, such as strontium loaded coatings, are accumulating preliminary clinical evidence (Diez-Escudero and Hailer, 2021; Guan et al., 2024; Zhao et al., 2020). In the field of wound care, products such as silver dressings have been proven to have value in chronic wound management through multiple studies (Luo et al., 2022). However, the vast majority of intelligent response and multifunctional coatings are still in the preclinical or early clinical stages, and their clinical translation faces core challenges such as a lack of long-term safety data, unclear regulatory pathways for complex products, significant differences in individualized efficacy, and insufficient evidence in health economics (Peng et al., 2025). Therefore, this review aims to provide a clear perspective for researchers in the field to connect laboratory innovation with clinical practical needs by integrating these clinical evidence and translational bottlenecks, and promote the development of next-generation coating technologies towards a more evidence-based and translational direction.

## 6 Challenges and outlook

Medical coatings, as the bridge between biomaterials and living systems, have evolved from initial barrier functions into functionally diverse, responsively intelligent precision engineering systems. In numerous fields such as orthopedics, cardiovascular, dentistry, drug delivery, and anti-infection, medical coatings have significantly enhanced the performance of medical devices and therapeutic outcomes. However, with increasingly complex medical demands and the advent of the era of precision medicine, this field faces unprecedented challenges and opportunities.

Firstly, medical coatings still struggle to achieve a balance between long-term stability and biocompatibility. In the complex physiological environment of the body, coating materials face multiple challenges such as protein adsorption, cell interactions, enzymatic degradation, and mechanical stress. Coating delamination, degradation, or structural failure not only leads to loss of function but may also trigger serious biological reactions. For example, in drug-eluting stents, premature degradation or abnormal delamination of the polymer coating may cause serious complications like late stent thrombosis (Giustino et al., 2022; Stone et al., 2005). Simultaneously, the biocompatibility assessment of coating materials needs to go beyond traditional cytotoxicity, considering the metabolic pathways of their degradation products during long-term implantation, immunogenicity, and potential impacts on specific tissues and organs. Recently, although some new coatings exhibit excellent biological performance, the potential release, migration, and accumulation effects of their nanoparticles in organs still require more systematic long-term safety evaluations (Chen et al., 2023). Secondly, because the human body’s response mechanisms to different types of implants vary, this places highly specific requirements on coating design. At the vascular interface, a balance between rapid endothelialization and anticoagulant function needs to be achieved; at the bone interface, the dynamic process of osteoblast activity and osteoclast resorption needs coordination; at the neural interface, guided axonal growth and inhibition of glial scar formation are essential. Tissue specificity requires coatings to simultaneously provide appropriate biochemical signals, topological structures, and mechanical properties. However, most current coatings still struggle to precisely mimic this complex biointerface, especially having limited capabilities in dynamic regulation. Once implanted, the coating’s performance is basically fixed, unable to adapt to the changing needs of different stages in the tissue healing process. Finally, the clinical translation of medical coating technology faces multiple obstacles. At the manufacturing process level, successful coating technologies in the laboratory are often difficult to scale up for large-scale, low-cost, high-consistency industrial production. Characterization methods and quality standards for complex coating systems are not yet perfected, lacking effective online monitoring and terminal testing methods. Furthermore, regulatory approval paths are more complex, especially for composite coatings incorporating bioactive molecules or cells, which often need to meet dual requirements for both medical devices and pharmaceuticals, greatly increasing the uncertainty and time cost of approval.

Future medical coatings will transcend single functions, developing towards multifunctional integration. An ideal coating system can simultaneously possess multiple functions such as anti-infection, anti-inflammatory, promoting tissue regeneration, and inhibiting foreign body reactions, and these functions can achieve temporal synergy. For example, in orthopedic implant coatings, a multi-stage functional sequence can be designed: rapid release of antibacterial agents in the early stage to prevent infection, sustained release of anti-inflammatory molecules in the mid-term to reduce foreign body reactions, and slow release of osteogenic factors in the later stage to promote osseointegration. Achieving precise temporal control and spatial distribution regulation of multiple therapeutic functions through material design and engineering innovation will be an important breakthrough direction for coating technology. Meanwhile, medical coatings will develop towards greater intelligence, capable of sensing changes in the internal microenvironment and responding in real-time. Smart coatings can be designed to respond to specific biomarkers (e.g., enzymes, pH, reactive oxygen species) or external stimuli (e.g., light, magnetism, ultrasound), achieving on-demand drug release or dynamic regulation of surface properties. For example, at the implantation site after tumor resection, pH-responsive coatings can be designed to release chemotherapeutic drugs only under the acidic conditions of the tumor microenvironment; in diabetes-related treatments, glucose-responsive coatings can be developed to automatically regulate insulin release based on blood glucose levels. These intelligent systems will make treatment more precise and efficient while minimizing side effects.

In summary, medical coating technology is in a significant transition period from “passive compatibility” to “active regulation,” from “single function” to “intelligent integration.” Although severe challenges remain in long-term stability, biocompatibility, clinical translation, and personalized application, through the synergistic innovation of smart material design, multifunctional integration, green manufacturing, computational methods, and personalized solutions, this field is demonstrating unprecedented development potential. With breakthroughs in basic science and advancements in engineering technology, the next-generation of medical coatings will undoubtedly become more precise, intelligent, and safe, becoming a true “intelligent interface” connecting biomaterials and living systems, providing key technological support for the era of precision medicine, and ultimately benefiting a wide range of patients. The field of medical coatings is transitioning decisively from a materials-centric endeavor to a discipline of dynamic biointerface engineering. The principal path forward lies in the convergence of several frontiers: the development of multifunctional and synergistic coatings that perform in biological sequences; the creation of smart systems that move beyond simple stimulus-response toward closed-loop, adaptive therapy; and the faithful mimicry of native tissue through biomechanical and biochemical cues. Addressing these priorities will be pivotal in realizing the promise of next-generation coatings as intelligent partners in healing and regeneration.

By charting the evolution of coatings from static, passive layers to intelligent, responsive systems, this review provides an integrative perspective that highlights the paradigm shift towards precision biomedicine, a holistic viewpoint that underscores the transformative potential of the field beyond incremental improvements.
